# Syllables per second versus seconds per syllable when measuring reading speed

**DOI:** 10.3389/fpsyg.2012.00518

**Published:** 2012-11-16

**Authors:** Alessio Toraldo, Maria Luisa Lorusso

**Affiliations:** ^1^DSU, Section of Psychology, University of PaviaPavia, Italy; ^2^Cognitive Psychology and Neuropsychology Unit, Scientific Institute “E. Medea”, Bosisio PariniLecco, Italy

## Syll/sec and sec/syll scores are not equivalent, even if they come from the same test

The diagnosis of developmental dyslexia requires measures of reading ability to be at least two standard deviations (SD) below age mean, encompassing accuracy, speed, and comprehension (ICD-10, WHO, 1992). Reading speed is typically measured in either seconds per syllable (sec/syll) or syllables per second (syll/sec). Although syll/sec seems to be more common in text or sentence reading tests while sec/syll seems to be preferred for word and non-word reading tests, the two scores are often provided interchangeably for the *same* test. Italian standardized tests offer a clear example: the “official” text-reading test (Cornoldi et al., 1986, 2011) and the widely used Battery for the Diagnosis of Reading and Spelling Disabilities (Sartori et al., 1995, 2007) provide norms measured as either syll/sec or total reading time (i.e., sec/syll multiplied by a constant—the overall number of syllables), depending on the edition or the specific version of the text.

**Figure 1:**
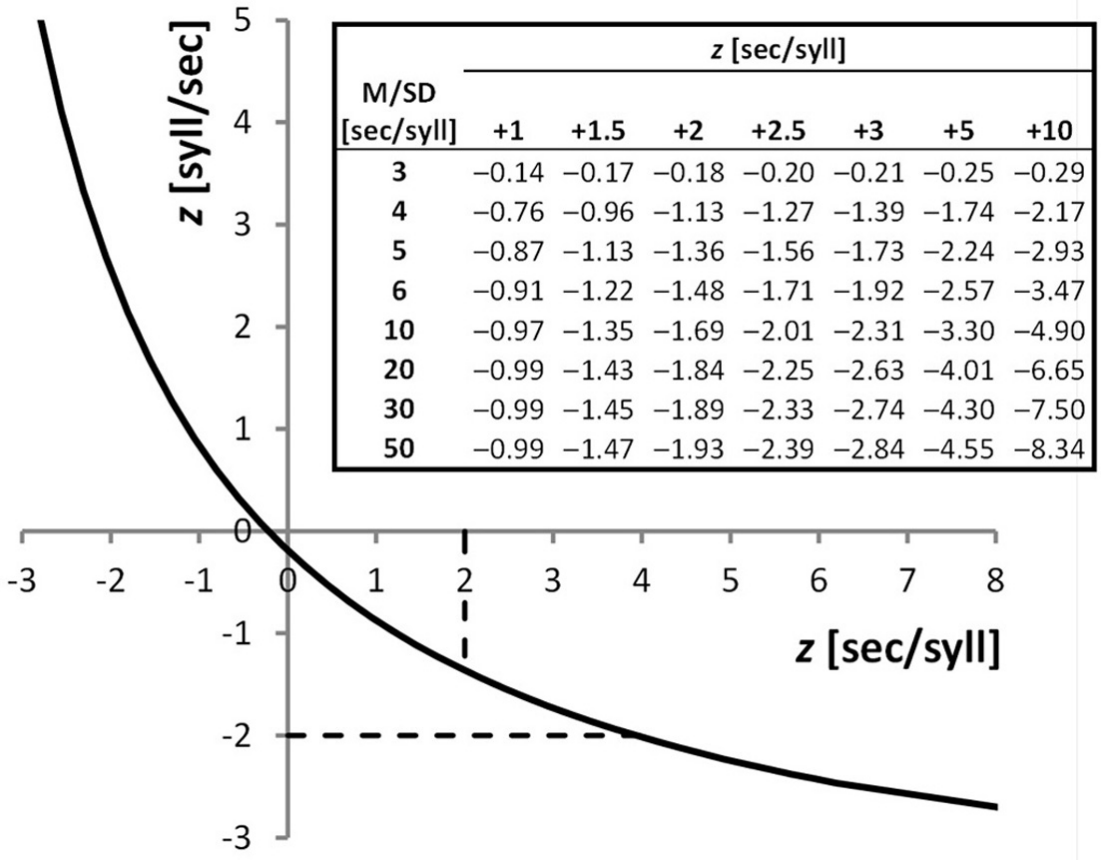
**Plot:** Hyperbolic function linking sec/syll and syll/sec from a same test performance; *z*-scores from sec/syll (obtained from a Monte Carlo simulation with Mean/SD ratio = 5) are plotted against *z*-scores from syll/sec. Dashed lines show normality cut-offs (+2 SDs for sec/syll, −2 SDs for syll/sec), which are clearly discrepant. Performances falling in the region of the curve between the intersections with the dashed lines (*z*-sec/syll values from +2 to +3.945) will be considered pathological using sec/syll, and normal using syll/sec. **Table**: *z*-scores from the syll/sec scale as a function of *z*-scores from the sec/syll scale (different columns) and of the distribution of sec/syll scores (rows). Normal distributions of sec/syll scores were simulated (*N* = 20,000), with different Mean/SD ratios (3–50). For instance, a child whose *z*-score from sec/syll is +2, with the normative sample having a Mean/SD ratio of 6, has a *z*-score from syll/sec of −1.48.

The purpose of our work is to discuss the use of syll/sec and sec/syll when these are derived from a same, single test. Intuitively, in this situation syll/sec and sec/syll scores have exactly the same meaning—the underlying numbers, of syllables and of seconds, are the same, with the former being the numerator and the latter the denominator of the fraction or vice-versa. Indeed they are treated as generally equivalent. When reading speed is taken as an inclusion criterion for research studies, most papers do not even specify whether a sec/syll or syll/sec score was used. However, sec/syll and syll/sec scores from a same test, as any pair of mutual reciprocals, are non-linearly related to each other, with the curve being a hyperbolic function (Figure 1). This has a number of unsuspected consequences. First, diagnostic decisions can be completely different if one relies on one or the other score. For instance, a child whose sec/syll score is in the pathological range (*z* = +2.00) would be placed in the borderline range, close to normal, by using syll/sec (*z* = −1.36). Similarly, a syll/sec *z-*score of −1.73 which is borderline, couples with a sec/syll *z-*score of +3.00 indicating severe deficit (Figure 1, top-right table, Mean/SD ratio = 5; these data are from a Monte Carlo study reported in the next paragraph). In the range of gross impairment, discrepancies widen more and more, and can become huge. Indeed, syll/sec is bounded in the deficit direction, with the scale being extremely “compressed” in that region. By contrast, sec/syll has no limit in the pathology direction. Thus, a child who improves from 10 to 5 sec/syll (with normal children having Mean = 0.5 and SD = 0.1), will have a “huge” change of 50 *z*-units on the sec/syll scale, and a “minuscule” improvement of only 0.2 *z*-units in the compressed syll/sec scale.

One might wonder exactly how large the expected sec/syll-syll/sec discrepancies are in a given real dataset. To answer this question we performed an extensive Monte Carlo simulation study (Figure 1, top-right table). The expected discrepancy is a function of the ratio between mean and SD of the standardization sample on one of the two scores. If the sample has a mean that is many times bigger than its internal variability, the discrepancy between the two diagnostic outcomes will in general be relatively small. Unfortunately, this is not the case in real standardization samples: mean values tend to be only 5–10 times as large as the SD (Cornoldi et al., 1986, 2011; Sartori et al., 1995, 2007; Lewandowski et al., 2003). So discrepancies in real data are likely to be sizeable. The reader can use our table in Figure 1 to have an approximate idea of the discrepancy range of any given data set.

Another kind of serious discrepancies can emerge when *differences of differences* (interaction terms in factorial designs) are considered. Two differences might well be equal on the syll/sec scale: a child might improve from *z* = −4 to *z* = −3 and another child from −2 to −1, but, because of the non-linear function relating the two scores, those improvements will in general be different on the sec/syll scale. Interaction terms are critical in intervention studies, which compare the effects of two or more types of treatment in different groups of children who are tested before and after treatment. It is well possible that an interaction is highly significant with one score, and non-significant with the other score, with obvious consequences on the conclusions of those studies.

In summary, when one derives sec/syll and syll/sec scores from a same test performance, the pronounced non-linearity of the function relating them can produce important discrepancies between results obtained with each score, both in clinical practice and in experimental research. This problem has been widely overlooked in the relevant literature, probably because the two scores are believed to be equivalent insofar as they carry the same information.

It is important to note that sec/syll and syll/sec are just one example of mutual reciprocals (another example is: frequency and period). All ratios between two measures *x* and *y* will pose exactly the same problems: *x/y* and *y/x* will give discrepant results, no matter what *x* and *y* are (times, lengths, weights, counts, etc.).

## Solutions

Which score should then be used? The answer to this question depends on the specific purpose one has. We will discuss five different purposes (Questions A–E, in separate sections below), which are the most common in clinical and experimental work. Other solutions will be listed in a further section.

### Question A: simple diagnostic criterion. is a child's performance normal or pathological?

A standard answer to this question is the use of percentiles, which are exactly equivalent for both sec/syll and syll/sec. Taking the 5th percentile of the syll/sec scale as a cut-off for normality is the same thing as taking the 95th percentile of the sec/syll scale. Similar reasoning can be applied to any other percentile-based classification or to non-parametric tolerance limits (Somerville, 1958).

### Question B: comparison between different groups of children, e.g., children with different associated disorders

The question whether two or more groups of children differ, can satisfactorily be answered by using non-parametric statistical tests (e.g., Mann–Whitney, Kruskal–Wallis, etc.). These again will give identical results from both syll/sec and sec/syll, because both scores provide the same ordering of subjects.

### Question C: between-test comparison. e.g., is a child's performance on words better than his/her performance on non-words?

This question can be rephrased as: “how severe is the deficit with Words with respect to the deficit with Nonwords?” A shared scale is needed that allows one to compare deficit severity across different stimulus types. Most typically, *z*-scores are obtained from Word and Non-word performances, and then compared, under the (ideal) assumption that equally severe underlying deficits will lead to equal *z*-scores. Hence, a logical way to choose between syll/sec and sec/syll would be to select the one which comes closer to satisfying such an assumption. This is, currently, a very difficult task. Indeed, we would need to know the precise shape of the functions relating the amount of underlying dyslexic deficit and the empirical reading performance—i.e., the “resource-performance” functions (Shallice, 1988). This in turn, would require a reliable and complete *neuro-cognitive* model of reading ability (see e.g., Kyngdon, 2011) as well as of its impairment. Currently, there is no theory of dyslexia which is complete and detailed enough to allow deduction of the resource-performance curves. So, there is no safe way to decide which score, syll/sec or sec/syll, is to be preferred. Therefore, when one wishes to compare performances on different tests, our suggestion is to present analyses from both scores.

### Question D: comparison across time. has a child's performance improved significantly (e.g., more than that of an untreated control sample)?

This question requires to compare the change in performance in one child, with respect to the changes observed in a control sample. However, the child's scores may lie in a different region of the scale than those of the control sample—so the assumption that score differences can be compared across the whole scale needs to be made. In other words, the change from (e.g.) 0.5 to 1 syll/sec and the change from 1 to 1.5 syll/sec must be assumed to reflect an identical improvement in the underlying deficit. This means that the resource-performance function should be linear. The natural solution would then be to choose the score, syll/sec or sec/syll, whose resource-performance function is closer to linear. However, as we already discussed, there are currently no theories of dyslexia that are complete and detailed enough for deriving resource-performance functions. So again, there is currently no way to choose between syll/sec and sec/syll on these grounds. Hence reporting results from both scores is advisable also in this case.

### Question E: comparing changes across time between different groups of children. e.g., have two groups, which received different treatments, improved to a same degree?

To compare changes of performance between different groups of children, one needs to assume that score differences have the same meaning all across the scale. So the same reasoning we proposed for the previous Question (D), holds here as well.

### Other solutions

Even though the ideal solution to the sec/syll-syll/sec dilemma for Questions C, D, E above would be to possess deep knowledge of resource-performance functions, there are other solutions which provide some practical advantages (albeit not fully theoretically justified). These are:

1. To use logarithmic transformations: *log*(sec/syll) and *log*(syll/sec) are entirely equivalent, insofar as they are linearly related to each other (Sarle, 1995; Martelli et al., 2008). However, this move simply makes the two scores agree with each other, without answering the question “which of them *meaningfully* reflects deficit severity?”
2. To choose the score whose distribution is closer to the Normal. That would make percentiles correspond to *z*-scores according to standard Normal distribution tables. However, percentiles do not allow one to tell apart different performance levels in the range of severe deficit (e.g., Tressoldi and Vio, 2007). A possible solution to this problem is to exploit the score distribution of impaired subjects instead of that of normal subjects (see Huber et al., 1983), but such data are rarely available and differ across age groups.
3. To choose sec/syll, because it provides a wider range of *z*-scores in the pathology domain (Stella, 2006). Indeed, if one uses the syll/sec scale, which is very compressed in the direction of pathology, even clinically significant improvements like a *halving* of the time taken to read a passage, would be reduced to a small fraction of a *z*-unit. Since most experts in both the clinical and the scientific community reason in terms of *z-units*, sec/syll scores could be preferred in order to avoid under-estimation of clinically significant changes.

## Conclusion

The puzzle we dealt with in this opinion paper is that analyses carried out using syll/sec vs. sec/syll scores which were obtained from a *same* reading performance, will necessarily be incongruent because of the non-linearity of the function relating them. This can have all sorts of undesirable consequences: studies using different scores may support different theories of dyslexia; dyslexia classifications may change according to the score used; diagnosis of dyslexia can vary according to the chosen score—an important issue to clinicians, given that such a diagnosis can provide access to treatment and to other facilities that are reserved to dyslexic individuals, at school, in occupational contexts and in social services.

We suggested the following solutions. Provided that *percentiles* are used, sec/syll and syll/sec can be used indifferently in dyslexia diagnosis. If, however, the clinician wishes to use *z*-scores as prescribed by diagnostic manuals, s/he should be aware that choosing syll/sec scores will produce less inclusive criteria (i.e., fewer children will have *z*-scores below −2 SDs and thus be diagnosed as dyslexic). Sec/syll and syll/sec are also equivalent if one is using non-parametric statistical tests to compare the performance of different dyslexic subgroups. When comparing different tests (e.g., Words vs. Non-words), or when comparing the changes across time in different children, one may report *both* sec/syll and syll/sec analyses, because there is currently no way to decide which score (if any) satisfies the required theoretical assumptions. Nonetheless, sec/syll could be preferred in clinical activity because, in a community where *z*-score transformation is widely accepted, it provides a better grasp of clinically significant improvement.
